# Supplementary material for: Systematic review of the uncertainty of coral reef futures under climate change
Source: Nat Commun. 2024 Mar 12;15:2224. doi: 10.1038/s41467-024-46255-2 (PMC10933488; doi:10.1038/s41467-024-46255-2)
Supplement: Supplementary file 1 — Supplementary Information [file 41467_2024_46255_MOESM1_ESM.pdf]

Supplementary Information for

Systematic review of the uncertainty of coral reef futures under climate change

Author list

Shannon G. Klein[1,2,3], Cassandra Roch[1,2,3], Carlos M. Duarte[1,2,3]

Affiliations

1 Marine Science Program, Biological and Environmental Science and Engineering Division (BESE), King Abdullah University of Science and Technology (KAUST), Thuwal 23955-6900, Kingdom of Saudi Arabia

2 Red Sea Research Center (RSRC), King Abdullah University of Science and Technology (KAUST), Thuwal 23955-6900, Kingdom of Saudi Arabia

3 Computational Bioscience Research Center (CBRC), King Abdullah University of Science and Technology (KAUST), Thuwal 23955-6900, Kingdom of Saudi Arabia

Corresponding Author

shannon.klein@kaust.edu.sa

carlos.duarte@kaust.edu.sa

Supplementary Figures

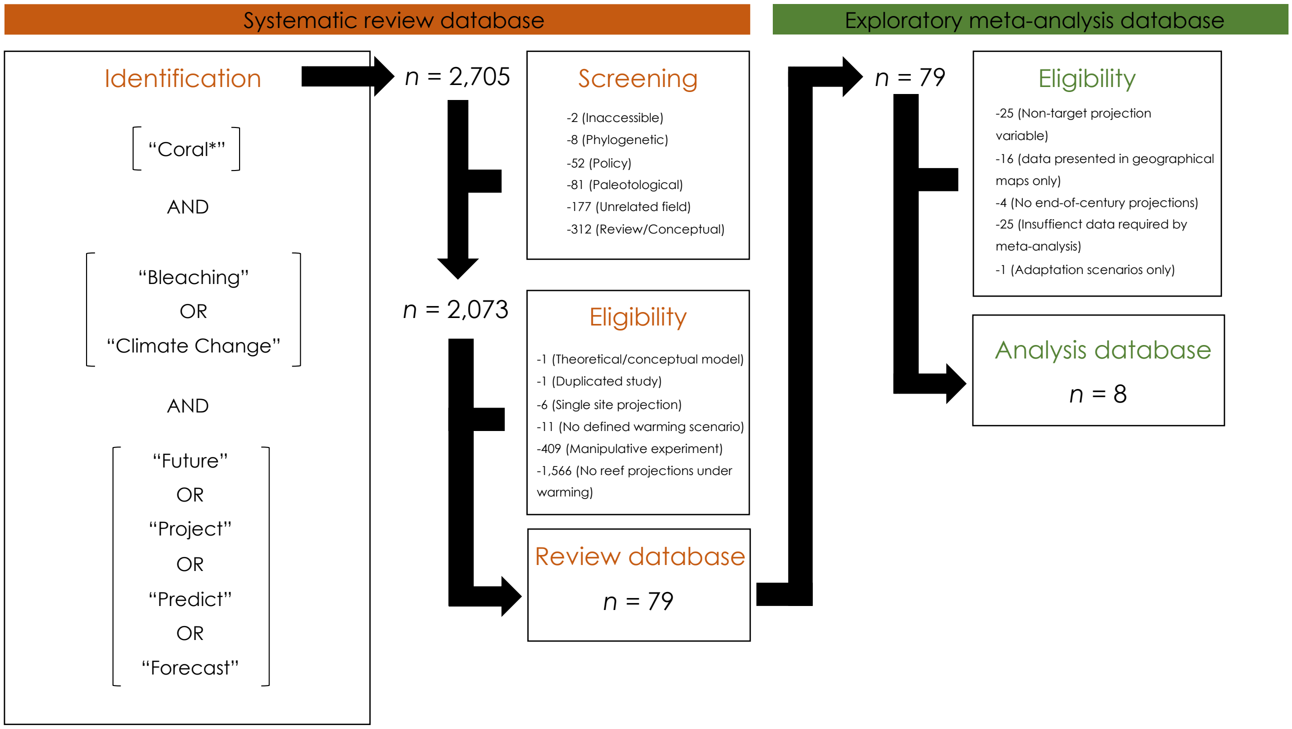

Supplementary Figure 1

Flowchart for the publication selection process based on PRISMA (Preferred Reporting Items for Meta-analyses) guidelines. Flowchart in orange depicts the selection process followed to include publications in the systematic review. Flowchart in green shows the selection process used to identify publications with extractable data for the exploratory meta-analysis (see Methods). Chart format is based on ref[1]. Search string listed in 'Identification' panel used Boolean logic and was used in the Web of Science (WoS) database.

Supplementary Tables

Supplementary Table 1

Distribution of studies across major approach categories at regional and geographical scales. Numbers in parentheses represent the number of studies within each category that used threshold metrics to formulate projections (see Methods for details). The asterisk represents one study that compared two separate modelling approaches, one of which used a threshold metric. See Supplementary Data 1 & 2 for extensive list of articles, spatial scale, and geographic focus.

	‘Excess heat’ threshold	Population dynamics	SDMs/Niche	Eco-Evolutionary	Meta-syntheses	Other	Total
Regional	9 (9)	17 (6*)	10 (2)	7 (3)	0 (0)	4 (1)	**47** (21)
Global	16 (16)	1 (1)	8 (1)	3 (2)	4 (1)	0 (0)	**32** (21)
Total	**25** (25)	**18** (7*)	**18** (3)	**10** (5)	**4** (1)	**4** (1)	**79** (42)

Supplementary Table 2

Number of studies that investigated the effect of additional pressures when forecasting the future effects of warming on coral reefs. See Supplementary Data 1 & 2 for extensive list of articles and focal stressor(s) examined.

	Ocean acidification	Pollution	Cyclone/ Hurricanes	Fishing pressure	Dissolved O_2	Sea level rise	Pest species	Pathogens
Threshold	14	5	4	2	2	1	1	1
Variable	14	11	8	2	1	0	0	0
Total	**28**	**16**	**12**	**4**	**3**	**1**	**1**	**1**

Supplementary Methods: Selection criteria for the meta-analysis

The following criteria were applied to select studies for inclusion in the exploratory meta-analysis:

1. The studies had to report model outputs as percentage or fractions of reef cells at risk, percentage of coral cover, or percentage change in habitat suitability/availability.
2. The studies had to clearly report the units of the model outputs, the number of simulations used, and provide a measure of uncertainty, including their units.
3. In cases where multiple distinct 'scenarios' (such as different geographic locations, coral species, or coral growth forms) were assessed within the same article, each scenario was treated as an individual observation and entered into the database as a separate row.
4. The focus of the analysis was on simulated responses of coral reefs to projected climate scenarios without considering adaptation. Accordingly, scenarios that incorporated thermal adaptation into the model were excluded from the analysis.
5. Data were extracted from studies that clearly described the future scenarios examined and projected coral reef responses at the end-of-century.
6. Data extraction was limited to cases where the baseline period fell between 2000 and 2015. In instances where baseline estimates could be extracted from graphs, they were standardized to the year 2010.

In total, out of the initial pool of 79 studies, eight studies were identified as having suitable quantitative data for inclusion in our analysis.

In this study, we computed effect sizes for three ecological metrics: percentage of coral cover, percentage of reefs at risk, and percentage of habitat change. To ensure consistent representation of positive or negative ecological impacts, we standardized the effect sizes by inverting the sign of metrics where necessary. For example, we reversed the sign for the 'percentage of reefs at risk' metric to correctly indicate a negative effect. This approach ensures a uniform and ecologically meaningful interpretation of the effect sizes across all metrics.

Supplementary References

1 Page, M. J. *et al.* The PRISMA 2020 statement: an updated guideline for reporting systematic reviews. *BMJ* **372**, n71, doi:10.1136/bmj.n71 (2021).

2 Khalil, I., Muslim, A. M., Hossain, M. S. & Atkinson, P. M. Modelling and forecasting the effects of increasing sea surface temperature on coral bleaching in the Indo-Pacific region. *International Journal of Remote Sensing* **44**, 194-216 (2023).

3 Abe, H., Kumagai, N. H. & Yamano, H. Priority coral conservation areas under global warming in the Amami Islands, Southern Japan. *Coral Reefs* **41**, 1637-1650 (2022).

4 Sully, S., Hodgson, G. & van Woesik, R. Present and future bright and dark spots for coral reefs through climate change. *Global Change Biology* **28**, 4509-4522, doi:https://doi.org/10.1111/gcb.16083 (2022).

5 DeFilippo, L. B. *et al.* Assessing the potential for demographic restoration and assisted evolution to build climate resilience in coral reefs. *Ecological applications* **32**, e2650 (2022).

6 Holstein, D. M., Smith, T. B., van Hooidonk, R. & Paris, C. B. Predicting coral metapopulation decline in a changing thermal environment. *Coral Reefs* **41**, 961-972, doi:10.1007/s00338-022-02252-9 (2022).

7 Raharinirina, N. A., Acevedo-Trejos, E. & Merico, A. Modelling the acclimation capacity of coral reefs to a warming ocean. *PLOS Computational Biology* **18**, e1010099 (2022).

8 Chollett, I. *et al.* Planning for resilience: Incorporating scenario and model uncertainty and trade-offs when prioritizing management of climate refugia. *Global Change Biology* **28**, 4054-4068 (2022).

9 Setter, R. O., Franklin, E. C. & Mora, C. Co-occurring anthropogenic stressors reduce the timeframe of environmental viability for the world's coral reefs. *PLOS Biology* **20**, e3001821, doi:10.1371/journal.pbio.3001821 (2022).

10 McWhorter, J. K., Halloran, P. R., Roff, G., Skirving, W. J. & Mumby, P. J. Climate refugia on the Great Barrier Reef fail when global warming exceeds 3° C. *Global Change Biology* **28**, 5768-5780 (2022).

11 Kalmus, P., Ekanayaka, A., Kang, E., Baird, M. & Gierach, M. Past the precipice? Projected coral habitability under global heating. *Earth's Future* **10**, e2021EF002608 (2022).

12 McWhorter, J. K. *et al.* The importance of 1.5°C warming for the Great Barrier Reef. *Global Change Biology* **28**, 1332-1341, doi:https://doi.org/10.1111/gcb.15994 (2022).

13 Klein, S. G. *et al.* Projecting coral responses to intensifying marine heatwaves under ocean acidification. *Global Change Biology* **n/a**, doi:https://doi.org/10.1111/gcb.15818 (2021).

14 Adam, A. A. *et al.* Diminishing potential for tropical reefs to function as coral diversity strongholds under climate change conditions. *Diversity and Distributions* **27**, 2245-2261 (2021).

15 Cant, J. *et al.* The projected degradation of subtropical coral assemblages by recurrent thermal stress. *Journal of Animal Ecology* **90**, 233-247 (2021).

16 Principe, S. C., Acosta, A. L., Andrade, J. E. & Lotufo, T. M. Predicted shifts in the distributions of Atlantic reef-building corals in the face of climate change. *Frontiers in Marine Science* **8**, 673086 (2021).

17 Strona, G. *et al.* Global tropical reef fish richness could decline by around half if corals are lost. *Proceedings of the Royal Society B* **288**, 20210274 (2021).
18 Bleuel, J., Pennino, M. G. & Longo, G. O. Coral distribution and bleaching vulnerability areas in Southwestern Atlantic under ocean warming. *Scientific Reports* **11**, 1-12 (2021).
19 Cornwall, C. E. *et al.* Global declines in coral reef calcium carbonate production under ocean acidification and warming. *Proceedings of the National Academy of Sciences* **118**, e2015265118, doi:doi:10.1073/pnas.2015265118 (2021).
20 McManus, L. C. *et al.* Evolution and connectivity influence the persistence and recovery of coral reefs under climate change in the Caribbean, Southwest Pacific, and Coral Triangle. *Global change biology* **27**, 4307-4321 (2021).
21 McClanahan, T. R. & Azali, M. K. Environmental Variability and Threshold Model's Predictions for Coral Reefs. *Frontiers in Marine Science* **8**, doi:10.3389/fmars.2021.778121 (2021).
22 Zuo, X. *et al.* Spatially Modeling the Synergistic Impacts of Global Warming and Sea-Level Rise on Coral Reefs in the South China Sea. *Remote Sensing* **13**, 2626 (2021).
23 McManus, L. C. *et al.* Extreme temperature events will drive coral decline in the Coral Triangle. *Global Change Biology* **26**, 2120-2133 (2020).
24 Rodriguez, L., García, J. J., Tuya, F. & Martínez, B. Environmental factors driving the distribution of the tropical coral Pavona varians: predictions under a climate change scenario. *Marine Ecology* **41**, 1-12 (2020).
25 Cacciapaglia, C. W. & van Woesik, R. Reduced carbon emissions and fishing pressure are both necessary for equatorial coral reefs to keep up with rising seas. *Ecography* **43**, 789-800, doi:https://doi.org/10.1111/ecog.04949 (2020).
26 Matz, M. V., Treml, E. A. & Haller, B. C. Estimating the potential for coral adaptation to global warming across the Indo-West Pacific. *Global Change Biology* (2020).
27 Kubicek, A., Breckling, B., Hoegh-Guldberg, O. & Reuter, H. Climate change drives trait-shifts in coral reef communities. *Scientific Reports* **9**, 3721, doi:10.1038/s41598-019-38962-4 (2019).
28 Rodriguez, L., Martínez, B. & Tuya, F. Atlantic corals under climate change: modelling distribution shifts to predict richness, phylogenetic structure and trait-diversity changes. *Biodiversity and Conservation* **28**, 3873-3890, doi:10.1007/s10531-019-01855-z (2019).
29 Jones, L. A. *et al.* Coupling of palaeontological and neontological reef coral data improves forecasts of biodiversity responses under global climatic change. *Royal Society Open Science* **6**, 182111 (2019).
30 Yan, H. *et al.* Regional coral growth responses to seawater warming in the South China Sea. *Science of the total environment* **670**, 595-605 (2019).
31 Woesik, R. v., Köksal, S., Ünal, A., Cacciapaglia, C. W. & Randall, C. J. Predicting coral dynamics through climate change. *Scientific reports* **8**, 17997 (2018).
32 Wolff, N. H., Mumby, P. J., Devlin, M. & Anthony, K. R. N. Vulnerability of the Great Barrier Reef to climate change and local pressures. *Global Change Biology* **24**, 1978-1991, doi:10.1111/gcb.14043 (2018).
33 Cacciapaglia, C. & van Woesik, R. Marine species distribution modelling and the effects of genetic isolation under climate change. *Journal of Biogeography* **45**, 154-163 (2018).
34 Kornder, N. A., Riegl, B. M. & Figueiredo, J. Thresholds and drivers of coral calcification responses to climate change. *Global Change Biology* **24**, 5084-5095, doi:https://doi.org/10.1111/gcb.14431 (2018).

35 Langlais, C. *et al.* Coral bleaching pathways under the control of regional temperature variability. *Nature Climate Change* **7**, 839-844 (2017).
36 Kendall, M. S., Poti, M. & Karnauskas, K. B. Climate change and larval transport in the ocean: fractional effects from physical and physiological factors. *Global Change Biology* **22**, 1532-1547, doi:https://doi.org/10.1111/gcb.13159 (2016).
37 Yara, Y. *et al.* Potential future coral habitats around Japan depend strongly on anthropogenic CO 2 emissions. *Aquatic biodiversity conservation and ecosystem services*, 41-56 (2016).
38 Van Hooidonk, R. *et al.* Local-scale projections of coral reef futures and implications of the Paris Agreement. *Scientific reports* **6**, 39666 (2016).
39 Schleussner, C.-F. *et al.* Differential climate impacts for policy-relevant limits to global warming: the case of 1.5 C and 2 C. *Earth system dynamics* **7**, 327-351 (2016).
40 Ainsworth, T. D. *et al.* Climate change disables coral bleaching protection on the Great Barrier Reef. *Science* **352**, 338-342, doi:doi:10.1126/science.aac7125 (2016).
41 Cooper, J. K., Spencer, M. & Bruno, J. F. Stochastic dynamics of a warmer Great Barrier Reef. *Ecology* **96**, 1802-1811 (2015).
42 Bozec, Y.-M. & Mumby, P. J. Synergistic impacts of global warming on the resilience of coral reefs. *Philosophical Transactions of the Royal Society B: Biological Sciences* **370**, 20130267 (2015).
43 Bozec, Y. M., Alvarez-Filip, L. & Mumby, P. J. The dynamics of architectural complexity on coral reefs under climate change. *Global change biology* **21**, 223-235 (2015).
44 van Hooidonk, R., Maynard, J. A., Liu, Y. & Lee, S. K. Downscaled projections of Caribbean coral bleaching that can inform conservation planning. *Global change biology* **21**, 3389-3401 (2015).
45 Kwiatkowski, L., Cox, P., Halloran, P. R., Mumby, P. J. & Wiltshire, A. J. Coral bleaching under unconventional scenarios of climate warming and ocean acidification. *Nature Climate Change* **5**, 777-781 (2015).
46 Maynard, J. *et al.* Projections of climate conditions that increase coral disease susceptibility and pathogen abundance and virulence. *Nature Climate Change* **5**, 688-694 (2015).
47 Descombes, P. *et al.* Forecasted coral reef decline in marine biodiversity hotspots under climate change. *Global Change Biology* **21**, 2479-2487 (2015).
48 Freeman, L. A. Robust performance of marginal Pacific coral reef habitats in future climate scenarios. *PLoS One* **10**, e0128875 (2015).
49 Cacciapaglia, C. & van Woesik, R. Reef-coral refugia in a rapidly changing ocean. *Global Change Biology* **21**, 2272-2282 (2015).
50 Mumby, P. J., Wolff, N. H., Bozec, Y.-M., Chollett, I. & Halloran, P. Operationalizing the Resilience of Coral Reefs in an Era of Climate Change. *Conservation Letters* **7**, 176-187, doi:https://doi.org/10.1111/conl.12047 (2014).
51 Yara, Y., Fujii, M., Yamano, H. & Yamanaka, Y. Projected coral bleaching in response to future sea surface temperature rises and the uncertainties among climate models. *Hydrobiologia* **733**, 19-29 (2014).
52 Logan, C. A., Dunne, J. P., Eakin, C. M. & Donner, S. D. Incorporating adaptive responses into future projections of coral bleaching. *Global Change Biology* **20**, 125-139 (2014).

53 van Hooidonk, R., Maynard, J. A., Manzello, D. & Planes, S. Opposite latitudinal gradients in projected ocean acidification and bleaching impacts on coral reefs. *Global Change Biology* **20**, 103-112, doi:https://doi.org/10.1111/gcb.12394 (2014).
54 Ortiz, J. C., González-Rivero, M. & Mumby, P. J. An ecosystem-level perspective on the host and symbiont traits needed to mitigate climate change impacts on Caribbean coral reefs. *Ecosystems* **17**, 1-13 (2014).
55 Lane, D. R. *et al.* Quantifying and valuing potential climate change impacts on coral reefs in the United States: Comparison of two scenarios. *PloS one* **8**, e82579 (2013).
56 Kennedy, E. V. *et al.* Avoiding coral reef functional collapse requires local and global action. *Current Biology* **23**, 912-918 (2013).
57 van Hooidonk, R., Maynard, J. A. & Planes, S. Temporary refugia for coral reefs in a warming world. *Nature Climate Change* **3**, 508-511, doi:10.1038/nclimate1829 (2013).
58 Frieler, K. *et al.* Limiting global warming to 2 C is unlikely to save most coral reefs. *Nature Climate Change* **3**, 165 (2013).
59 Ortiz, J. C., González-Rivero, M. & Mumby, P. J. Can a thermally tolerant symbiont improve the future of Caribbean coral reefs? *Global change biology* **19**, 273-281 (2013).
60 Freeman, L. A., Kleypas, J. A. & Miller, A. J. Coral reef habitat response to climate change scenarios. *PloS one* **8**, e82404 (2013).
61 Couce, E., Ridgwell, A. & Hendy, E. J. Future habitat suitability for coral reef ecosystems under global warming and ocean acidification. *Global Change Biology* **19**, 3592-3606, doi:https://doi.org/10.1111/gcb.12335 (2013).
62 Couce, E., Irvine, P. J., Gregoire, L., Ridgwell, A. & Hendy, E. Tropical coral reef habitat in a geoengineered, high-CO2 world. *Geophysical Research Letters* **40**, 1799-1805 (2013).
63 Wooldridge, S. A. *et al.* Safeguarding coastal coral communities on the central Great Barrier Reef (Australia) against climate change: realizable local and global actions. *Climatic Change* **112**, 945-961 (2012).
64 Meissner, K., Lippmann, T. & Sen Gupta, A. Large-scale stress factors affecting coral reefs: open ocean sea surface temperature and surface seawater aragonite saturation over the next 400 years. *Coral Reefs* **31**, 309-319 (2012).
65 van Hooidonk, R. & Huber, M. Effects of modeled tropical sea surface temperature variability on coral reef bleaching predictions. *Coral Reefs* **31**, 121-131, doi:10.1007/s00338-011-0825-4 (2012).
66 Teneva, L. *et al.* Predicting coral bleaching hotspots: the role of regional variability in thermal stress and potential adaptation rates. *Coral Reefs* **31**, 1-12 (2012).
67 Yara, Y. *et al.* Ocean acidification limits temperature-induced poleward expansion of coral habitats around Japan. *Biogeosciences* **9**, 4955-4968 (2012).
68 Edwards, H. J. *et al.* How much time can herbivore protection buy for coral reefs under realistic regimes of hurricanes and coral bleaching? *Global Change Biology* **17**, 2033-2048 (2011).
69 Anthony, K. R. N. *et al.* Ocean acidification and warming will lower coral reef resilience. *Global Change Biology* **17**, 1798-1808, doi:https://doi.org/10.1111/j.1365-2486.2010.02364.x (2011).
70 Hoegh-Guldberg, O. Coral reef ecosystems and anthropogenic climate change. *Regional Environmental Change* **11**, 215-227 (2011).

71 Hoeke, R. K., Jokiel, P. L., Buddemeier, R. W. & Brainard, R. E. Projected changes to growth and mortality of Hawaiian corals over the next 100 years. *PloS one* **6**, e18038 (2011).

72 McLeod, E. *et al.* Warming seas in the Coral Triangle: coral reef vulnerability and management implications. *Coastal Management* **38**, 518-539 (2010).

73 Baskett, M. L., Gaines, S. D. & Nisbet, R. M. Symbiont diversity may help coral reefs survive moderate climate change. *Ecological Applications* **19**, 3-17 (2009).

74 Vivekanandan, E., Ali, M. H., Jasper, B. & Rajagopalan, M. Vulnerability of corals to warming of the Indian seas: a projection for the 21st century. *Current Science*, 1654-1658 (2009).

75 Donner, S. D. Coping with commitment: projected thermal stress on coral reefs under different future scenarios. *PLoS One* **4**, e5712 (2009).

76 Buddemeier, R. W. *et al.* A modeling tool to evaluate regional coral reef responses to changes in climate and ocean chemistry. *Limnology and Oceanography: Methods* **6**, 395-411 (2008).

77 Donner, S. D., Skirving, W. J., Little, C. M., Oppenheimer, M. & Hoegh-Guldberg, O. Global assessment of coral bleaching and required rates of adaptation under climate change. *Global Change Biology* **11**, 2251-2265 (2005).

78 McNeil, B. I., Matear, R. J. & Barnes, D. J. Coral reef calcification and climate change: The effect of ocean warming. *Geophysical Research Letters* **31** (2004).

79 Guinotte, J., Buddemeier, R. & Kleypas, J. Future coral reef habitat marginality: temporal and spatial effects of climate change in the Pacific basin. *Coral reefs* **22**, 551-558 (2003).

80 Hoegh-Guldberg, O. Climate change, coral bleaching and the future of the world's coral reefs. *Marine and freshwater research* **50**, 839-866 (1999).
