# Supplementary material for: Systematic review of the uncertainty of coral reef futures under climate change
Source: Nat Commun. 2024 Mar 12;15:2224. doi: 10.1038/s41467-024-46255-2 (PMC10933488; doi:10.1038/s41467-024-46255-2)
Supplement: Supplementary file 3 — Description of Additional Supplementary Files [file 41467_2024_46255_MOESM3_ESM.pdf]

Description of Additional Supplementary Files

File name Supplementary Data 1

Description Summary table of main study characteristics for all articles included in the systematic review.

File name Supplementary Data 2

Description Comprehensive dataset capturing all study characteristics across all articles in the systematic review.

File name Supplementary Data 3

Description Individual scenario descriptions for the studies included in the effect size analysis.
