# Supplementary material for: Systematic review of the uncertainty of coral reef futures under climate change
Source: Nat Commun. 2024 Mar 12;15:2224. doi: 10.1038/s41467-024-46255-2 (PMC10933488; doi:10.1038/s41467-024-46255-2)
Supplement: Supplementary file 7 — Reporting Summary [file 41467_2024_46255_MOESM7_ESM.pdf]

nature portfolio

Corresponding author(s): Shannon G. Klein & Carlos M. Duarte

Last updated by author(s): Jan 23, 2024

Reporting Summary

Nature Portfolio wishes to improve the reproducibility of the work that we publish. This form provides structure for consistency and transparency in reporting. For further information on Nature Portfolio policies, see our Editorial Policies and the Editorial Policy Checklist.

Statistics

For all statistical analyses, confirm that the following items are present in the figure legend, table legend, main text, or Methods section.

n/a	Confirmed	
[ ]	[x]	The exact sample size (n) for each experimental group/condition, given as a discrete number and unit of measurement
[x]	[ ]	A statement on whether measurements were taken from distinct samples or whether the same sample was measured repeatedly
[x]	[ ]	The statistical test(s) used AND whether they are one- or two-sided<br>*Only common tests should be described solely by name; describe more complex techniques in the Methods section.*
[x]	[ ]	A description of all covariates tested
[x]	[ ]	A description of any assumptions or corrections, such as tests of normality and adjustment for multiple comparisons
[ ]	[x]	A full description of the statistical parameters including central tendency (e.g. means) or other basic estimates (e.g. regression coefficient) AND variation (e.g. standard deviation) or associated estimates of uncertainty (e.g. confidence intervals)
[x]	[ ]	For null hypothesis testing, the test statistic (e.g. F, t, r) with confidence intervals, effect sizes, degrees of freedom and P value noted<br>*Give P values as exact values whenever suitable.*
[x]	[ ]	For Bayesian analysis, information on the choice of priors and Markov chain Monte Carlo settings
[x]	[ ]	For hierarchical and complex designs, identification of the appropriate level for tests and full reporting of outcomes
[ ]	[x]	Estimates of effect sizes (e.g. Cohen's d, Pearson's r), indicating how they were calculated

Our web collection on statistics for biologists contains articles on many of the points above.

Software and code

Policy information about availability of computer code

Data collection	We searched the Thomson Reuters Web of Science database (http://www.webofknowledge.com) to identify studies projecting the impact of climate change on shallow tropical and sub-tropical coral reefs. The search was performed on March 6, 2023 and retrieved 2705 peer-reviewed articles. The final database consisted of 79 peer-reviewed articles published between 1999 and 2023. Data were extracted manually by two authors of the study and in some cases values were extracted using PlotDigitizer (plotdigitizer.com).
Data analysis	All effect size calculations were computed using the metafor package (version 4.2-0) in R (version 4.3.0).

For manuscripts utilizing custom algorithms or software that are central to the research but not yet described in published literature, software must be made available to editors and reviewers. We strongly encourage code deposition in a community repository (e.g. GitHub). See the Nature Portfolio guidelines for submitting code & software for further information.

Data

Policy information about availability of data

All manuscripts must include a data availability statement. This statement should provide the following information, where applicable:

- Accession codes, unique identifiers, or web links for publicly available datasets
- A description of any restrictions on data availability
- For clinical datasets or third party data, please ensure that the statement adheres to our policy

The source data supporting Figs. 1-5 are available in the Source Data file. Supplementary Data Files 1-3 provide a summary of all other data generated by this study, and the complete database is deposited in Dryad (https://doi.org/10.5061/dryad.4f4qrfjkp)

Research involving human participants, their data, or biological material

Policy information about studies with human participants or human data. See also policy information about sex, gender (identity/presentation), and sexual orientation and race, ethnicity and racism.

Reporting on sex and gender	N/A
Reporting on race, ethnicity, or other socially relevant groupings	N/A
Population characteristics	N/A
Recruitment	N/A
Ethics oversight	N/A

Note that full information on the approval of the study protocol must also be provided in the manuscript.

Field-specific reporting

Please select the one below that is the best fit for your research. If you are not sure, read the appropriate sections before making your selection.

☐ Life sciences ☐ Behavioural & social sciences ☒ Ecological, evolutionary & environmental sciences

For a reference copy of the document with all sections, see nature.com/documents/nr-reporting-summary-flat.pdf

Ecological, evolutionary & environmental sciences study design

All studies must disclose on these points even when the disclosure is negative.

Study description	We conducted a systematic review of 79 articles published between 1999 and 2023. We extracted key characteristics of each study, including the focal variables simulated, model inputs, spatial, scale, and geographical area. We classified the models into five main methodologies, as defined in the paper. Among the initial pool of 79 studies, eight studies were identified as containing quantitative data that could be extracted and compared in our analysis. For each study, we calculated Hedges' g effect sizes and variance for all individual scenarios/trajectories among three warming scenarios (39 scenarios in total, Supplementary Data 3).
Research sample	We searched the Thomson Reuters Web of Science database (http://www.webofknowledge.com) to identify studies projecting the impact of climate change on shallow tropical and sub-tropical coral reefs. The search was performed on March 6, 2023 and retrieved 2705 peer-reviewed articles. Our literature search strategy followed the guidelines of PRISMA (Preferred Reporting Items for Systematic Reviews and Meta-analyses). The final database consisted of 79 peer-reviewed articles published between 1999 and 2023.
Sampling strategy	Publications were selected based on the following criteria: (1) projections represented the responses of tropical and/or sub-tropical coral reefs to future levels of warming alone or in combination with any other drivers, (2) future emissions pathways and/or warming scenarios used to force the simulations were stated, and (3) projections were modeled across more than one reef site to be included in the database.
Data collection	S.G.K and C.R extracted the data from the published studies via manual value extraction, or extracted using PlotDigitizer (plotdigitizer.com).
Timing and spatial scale	The final database consisted of 79 peer-reviewed articles published between 1999 and 2023. The literature search was done on March 6, 2023.
Data exclusions	Publications generating projections of coral reef responses to future climate change that focused on individual reef sites were excluded to ensure a comparable synthesis with most other assessments at regional and global scales
Reproducibility	S.G.K and C.R independently verified the accuracy and reproducibility of data extractions for ~20% of the published articles. All data

	generated by this study are made available in supplementary data files and in the Dryad online repository.
Randomization	The list of published articles retrieved by the Web of Science database were randomized and randomly allocated to each extractor.
Blinding	If one extractor was an author of a published study in the database, the study was allocated to the other extractor. Any other form of blinding was not possible since the authors are listed on each published article.

Did the study involve field work? ☐ Yes ☒ No

Reporting for specific materials, systems and methods

We require information from authors about some types of materials, experimental systems and methods used in many studies. Here, indicate whether each material, system or method listed is relevant to your study. If you are not sure if a list item applies to your research, read the appropriate section before selecting a response.

Materials & experimental systems

n/a	Involved in the study
☒	☐ Antibodies
☒	☐ Eukaryotic cell lines
☒	☐ Palaeontology and archaeology
☒	☐ Animals and other organisms
☒	☐ Clinical data
☒	☐ Dual use research of concern
☒	☐ Plants

Methods

n/a	Involved in the study
☒	☐ ChIP-seq
☒	☐ Flow cytometry
☒	☐ MRI-based neuroimaging

Plants

Seed stocks	N/A
Novel plant genotypes	N/A
Authentication	N/A
